# The structure of bradyzoite-specific enolase from *Toxoplasma gondii* reveals insights into its dual cytoplasmic and nuclear functions

**DOI:** 10.1107/S1399004714026479

**Published:** 2015-02-26

**Authors:** Jiapeng Ruan, Thomas Mouveaux, Samuel H. Light, George Minasov, Wayne F. Anderson, Stanislas Tomavo, Huân M. Ngô

**Affiliations:** a Center for Structural Genomics of Infectious Diseases, Northwestern University, 320 E. Superior Street, Morton 7-601, Chicago, IL 60611, USA; b Center for Infection and Immunity of Lille, CNRS UMR 8204, INSERM U1019, Institut Pasteur de Lille, Université Lille Nord de France, France; c BrainMicro LLC, 21 Pendleton Street, New Haven, CT 06511, USA

**Keywords:** moonlighting protein, brain parasite, central nervous system, chronic infection, differentiation

## Abstract

In addition to catalyzing a central step in glycolysis, enolase assumes a remarkably diverse set of secondary functions in different organisms, including transcription regulation as documented for the oncogene c-Myc promoter-binding protein 1. The apicomplexan parasite *Toxoplasma gondii* differentially expresses two nuclear-localized, plant-like enolases: enolase 1 (TgENO1) in the latent bradyzoite cyst stage and enolase 2 (TgENO2) in the rapidly replicative tachyzoite stage. A 2.75 Å resolution crystal structure of bradyzoite enolase 1, the second structure to be reported of a bradyzoite-specific protein in *Toxoplasma*, captures an open conformational state and reveals that distinctive plant-like insertions are located on surface loops. The enolase 1 structure reveals that a unique residue, Glu164, in catalytic loop 2 may account for the lower activity of this cyst-stage isozyme. Recombinant TgENO1 specifically binds to a TTTTCT DNA motif present in the cyst matrix antigen 1 (TgMAG1) gene promoter as demonstrated by gel retardation. Furthermore, direct physical interactions of both nuclear TgENO1 and TgENO2 with the TgMAG1 gene promoter are demonstrated *in vivo* using chromatin immunoprecipitation (ChIP) assays. Structural and biochemical studies reveal that *T. gondii* enolase functions are multifaceted, including the coordination of gene regulation in parasitic stage development. Enolase 1 provides a potential lead in the design of drugs against *Toxoplasma* brain cysts.

## Abstract 2

The second crystal structure of a parasite protein preferentially enriched in the brain cyst of *T. gondii* has been solved at 2.75 Å resolution. Bradyzoite enolase 1 is reported to have differential functions as a glycolytic enzyme and a transcriptional regulator in bradyzoites.

## 1. Introduction   

Universally conserved across the three domains of life, enolase (EC 4.2.1.11) is an essential enzyme that catalyses the dehydration of 2-phospho-d-glycerate to phosphoenol­pyruvate in glycolysis and the reverse reaction in gluconeogenesis. In addition to playing a primary role in metabolism, enolases have evolved an impressive range of ‘moonlighting’ functions. For example, prokaryotic enolases have been shown to co-assemble into an RNA degradosome (Nurmohamed *et al.*, 2010 ▶; Chandran & Luisi, 2006 ▶), whereas eukaryotic enolases form a component of mitochondrial tRNA transport (Brandina *et al.*, 2006 ▶). Some mammalian enolases serve as surface receptors that bind human plasminogen and extracellular matrix and facilitate normal cellular migration (Bergmann *et al.*, 2005 ▶; Ghosh & Jacobs-Lorena, 2011 ▶). Interestingly, a number of pathogen enolases appear to have also evolved plasminogen-binding properties, allowing them to co-opt the plasmin pathway to promote the degradation of host tissue and pathogen invasion (Bergmann *et al.*, 2005 ▶; Ghosh *et al.*, 2011 ▶).

Several studies have reported that enolases localize to the nucleus, bind to DNA and act as transcription regulators. For example, *Arabidopsis thaliana* ENO2/LOS2 binds the promoter of the transcriptional factor STZ/ZAT10 and represses the gene transcription of cold response in plants (Lee *et al.*, 2002 ▶). Full-length human ENO1 (hENO1) and an alternative splice variant (hMPB1) bind to *cis*-promoters and repress the expression of key cell-cycle regulators such as c-Myc oncogene and cyclooxygenase 2 (COX-2) (Sedoris *et al.*, 2010 ▶; Hsu *et al.*, 2009 ▶). Although the DNA-binding potential is unknown in these organisms, enolase is localized to the nucleus of two protozoan parasites: *Plasmodium* (Pal- Bhowmick *et al.*, 2007 ▶, 2009 ▶) and *Entamoeba histolytica* (Tovy *et al.*, 2010 ▶).

The brain parasite *Toxoplasma gondii* provides a significant organism to examine the multifunctional roles of enolase. Infecting approximately one third of the global human population, this opportunistic apicomplexan parasite switches between two developmental stages in a human host: the pathogen proliferates in circulation and infected tissues as tachyzoites during primary infection, whereas it remains metabolically quiescent only in brain and muscle as tissue cysts containing the bradyzoites when the immune system has cleared the tachyzoites. Among *Toxoplasma*’s many pathologies, chronic brain infection has been implicated as a correlative factor in the development of neurological disorders such as schizo­affective disorder and suicidal risk (Flegr, 2013 ▶; Kannan & Pletnikov, 2012 ▶).

There are regrettably no drugs and vaccines to eliminate the parasitic cysts in the brains of chronically infected humans. Medicine discovery is further impeded by a relatively poor structural database for *Toxoplasma*. Of the >6000 *Toxoplasma* genes that are estimated from the *Toxoplasma* genome, structures have been determined for only ∼50 different proteins. Of these, ∼30 are enzymes that may be useful as potential drug target leads. To date, the structure of only one protein (a pseudo­aldolase) that is specifically up-regulated in the critical bradyzoite stage has been determined (Tonkin *et al.*, 2014 ▶).

*Toxoplasma* possesses two enolase isoforms that are differentially expressed in a stage-specific manner. Enolase 2 (TgENO2) has been shown to be specifically expressed in tachyzoites, whereas enolase 1 (TgENO1) is solely expressed in bradyzoites (Dzierszinski *et al.*, 1999 ▶). Biochemical studies have confirmed that both isoforms exhibit enolase activity but that the bradyzoite-specific TgENO1 has only one third of the enzymatic activity of TgENO2 (Dzierszinski *et al.*, 2001 ▶). Both TgENO1 and TgENO2 have been localized to the parasite nucleus and are regulated by different heat-stress promoter elements (Ferguson *et al.*, 2002 ▶; Mouveaux *et al.*, 2014 ▶). *In vivo*, both TgENO1 and TgENO2 bind to promoters and modulate gene expression (Mouveaux *et al.*, 2014 ▶). Nuclear TgENO2 is elevated in actively replicating tachyzoites and has been shown to bind to nuclear chromatin and occupy the promoter region of at least 242 genes. Tachyzoite TgENO2 has been shown to specifically bind the TTTTCT motif and activate promoters *in vitro* (Mouveaux *et al.*, 2014 ▶). Most significant of all, targeted disruption of bradyzoite TgENO1 reduces the cyst burden in the brain of chronically infected mice (Mouveaux *et al.*, 2014 ▶).

*Toxoplasma* enolases play important nuclear functions in parasite stage development (Coppin *et al.*, 2003 ▶; Kibe *et al.*, 2005 ▶), although the specific interaction of bradyzoite TgENO1 with a TTTTCT motif in gene promoters requires additional validation. In this study, we provide structural and biochemical data to provide insights into the multifunctional roles of enolases, with a special focus on the bradyzoite TgENO1.

## 2. Methods   

### 2.1. Plasmid and protein production   

Sequences and RNA-expression profiles of *Toxoplasma* enolases were extracted from ToxoDB (http://toxodb.org). The sequences of TgENO1 and TgENO2 were optimized for expression in *Escherichia coli*, synthesized and subcloned into the pCCK-N′-HisTEV vector (MCLAB, California, USA). Overnight cultures were prepared after the expression vectors had been transformed into BL21 (DE3) Magic *E. coli* cells. After inoculation with overnight culture, the cells were grown for 4 h at 37°C and protein expression was induced by the addition of 0.5 m*M* IPTG at 25°C. Harvested cells were suspended in 20 m*M* Tris–HCl, 500 m*M* NaCl, 10 m*M* imidazole buffer pH 8.3, sonicated in a water–ice bath (0–4°C) for 10 min and centrifuged for 10 min at 7000 rev min^−1^. The recombinant proteins were purified from the soluble cellular extract using an immobilized metal-affinity chromatography system with an Ni–NTA agarose column and gel filtration on a Sephadex G-25 column. Pure TgENO1 and TgENO2 eluted in a buffer consisting of 10 m*M* Tris–HCl pH 8.3, 500 m*M* NaCl, 5 m*M* β-mercaptoethanol. The protein was concentrated in the same buffer and used for crystal screening and *in vitro* studies.

### 2.2. Crystallization and X-ray data collection   

Sitting-drop crystallization experiments were set up using a 1:1 ratio of TgENO1 (7.0 mg ml^−1^) and reservoir solutions. The crystal used for structural studies was grown using a condition from the PACT screen (Qiagen) consisting of 0.1 *M* MMT (1:2:2 dl-malic acid:MES:Tris base) buffer pH 6.0 and 25% PEG 1500. The crystals were transferred to the reservoir condition for cryoprotection before being cooled in liquid nitrogen. Diffraction data were collected at 100 K on the Life Sciences Collaborative Access Team beamline at the Advanced Photon Source, Argonne, Illinois, USA. Diffraction images for the deposited structures are available at the CSGID website (http://www.csgid.org/csgid/pages/home).

### 2.3. Structure determination and refinement   

*HKL*-3000 was used for indexing, integration and scaling (Broennimann *et al.*, 2006 ▶). The structure was solved by molecular replacement in *Phaser* using the *Methanococcus jannaschii* enolase structure (PDB entry 2pa6; RIKEN Structural Genomics/Proteomics Initiative, unpublished work) as the starting model (McCoy *et al.*, 2005 ▶). The model was iteratively refined in *REFMAC* (Murshudov *et al.*, 2011 ▶) after undergoing manual corrections based on electron-density maps displayed in *Coot* (Emsley & Cowtan, 2004 ▶). Structure figures were prepared using *PyMOL* (v.1.5; Schrödinger), *PISA* (Xu *et al.*, 2008 ▶) and *LigPlot*+ v.1.3 (Laskowski & Swindells, 2011 ▶). Coordinates and structure factors have been deposited with the Protein Data Bank with accession number 3otr.

### 2.4. Biological source and DNA-binding assays   

Parasites were cultured in human foreskin fibroblasts, collected and nuclear extracts were obtained as described previously (Kibe *et al.*, 2005 ▶). The parasite nuclear extracts were aliquoted and stored at −80°C until use. Electrophoresis mobility shift assays (EMSAs) were performed using a band-shift assay kit (Thermo Scientific, France) following the manufacturer’s instructions. Competition experiments were completed as described previously (Mouveaux *et al.*, 2014 ▶). Chromatin immunoprecipitation (ChIP) analyses was performed as described by Olguin-Lamas *et al.* (2011 ▶) with slight modification. Briefly, chromatin from intracellular parasites (2 × 150 cm^3^ flasks) grown in HFF cells was cross-linked for 10 min with 1% formaldehyde at room temperature and purified as above. After cross-linking, the intracellular parasites were used to obtain chromatin extracts after sonication, yielding fragments of 500–1000 bp. Immunoprecipitations were performed using polyclonal anti-ENO1 antibodies (Dzierszinski *et al.*, 2001 ▶). Monoclonal or polyclonal anti-HA antibodies (Invitrogen) were also used. The ChIP was incubated at 4°C overnight and washed as described by Olguin-Lamas *et al.* (2011 ▶). The DNA was then subjected to proteinase K digestion for 2 h and purified using the Qiagen PCR purification kit (http://www.qiagen.com). As a negative control, pre-immune sera were used. ChIP products amplified by PCR using specific primers of the MAG1 gene were electrophoresed on agarose gels, stained with ethidium bromide and photographed using a UV-light scanner.

## 3. Results and discussion   

### 3.1. Stage-specific expression of *Toxoplasma* enolases   

RNA and protein expression data from the ToxoDB (*Toxoplasma* database; http://toxodb.org/toxo/; Gajria *et al.*, 2008 ▶) support previous reports of the stage-specific expression of the two enolase genes in the protozoan parasite *Toxoplasma* (Dzierszinski *et al.*, 1999 ▶). Although both enolases are expressed in all stages of the *Toxoplasma* life cycle, TgENO2 (TGME49_268850) is transcribed at its highest level in tachyzoites, whereas TgENO1 (TGME49_268860) expression is specifically upregulated in bradyzoites.

### 3.2. Structure of bradyzoite TgENO1   

**Figure 1:**
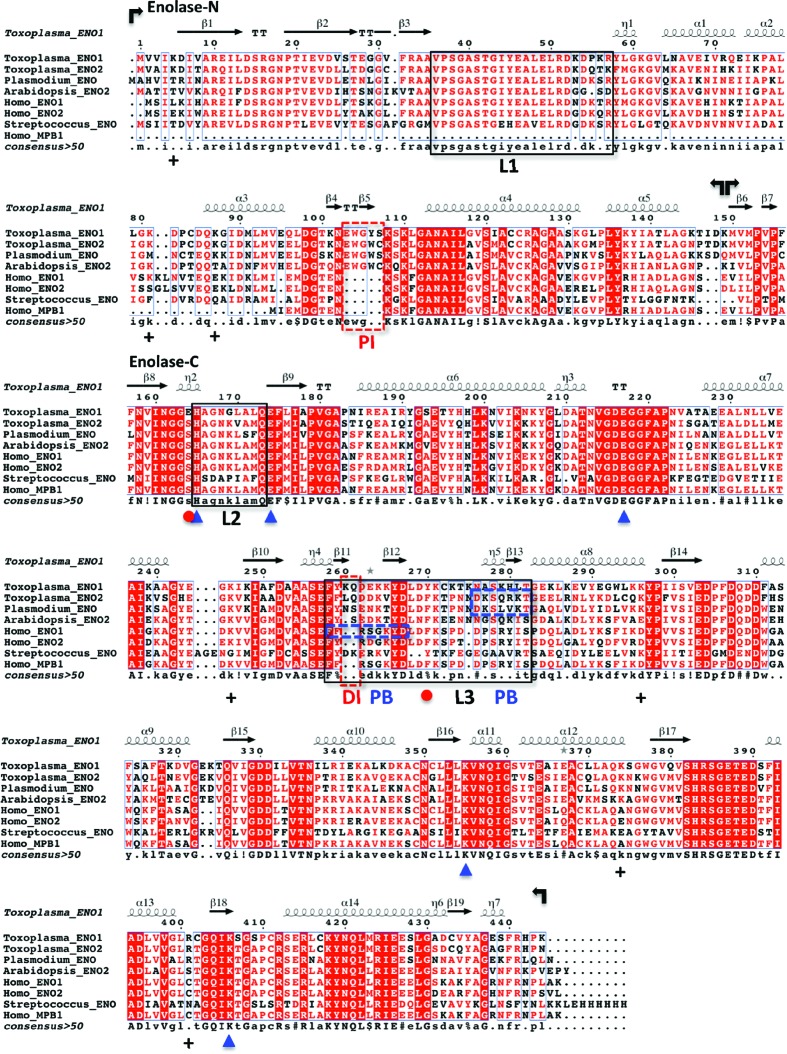
Secondary-structural alignment of enolases. Bacterial (*Streptococcus suis*), protozoan parasite (*Toxoplasma gondii* and *Plasmodium falciparum*), plant (*Arabidopsis thaliana*) and human enolases are aligned for comparison. The enolases are all nuclear-localized, with the exceptions of human ENO2 and *Streptococcus* ENO. Human MPB1 is an alternatively spliced form of ENO1 and is truncated at the N-terminal domain. The five conserved active-site residues (His165, Glu174, Glu217, Lys355 and Lys406) are marked by blue arrowheads. The atypical TgENO1 residue replacement E164S at the N-­terminus of loop 2 and the interacting residue Tyr270 in loop 3 are indicated by red dots. Conserved positively charged residues on the surface of transcription regulatory enolases are labelled with plus signs (+). Boxed regions are plant-like insertions (red dashes; DI, dipeptide insertion; PI, pentapeptide insertion), catalytic mobile loops (black; L, loop) and plasminogen-binding motifs (blue dashes; PB, plasminogen binding).

**Figure 2:**
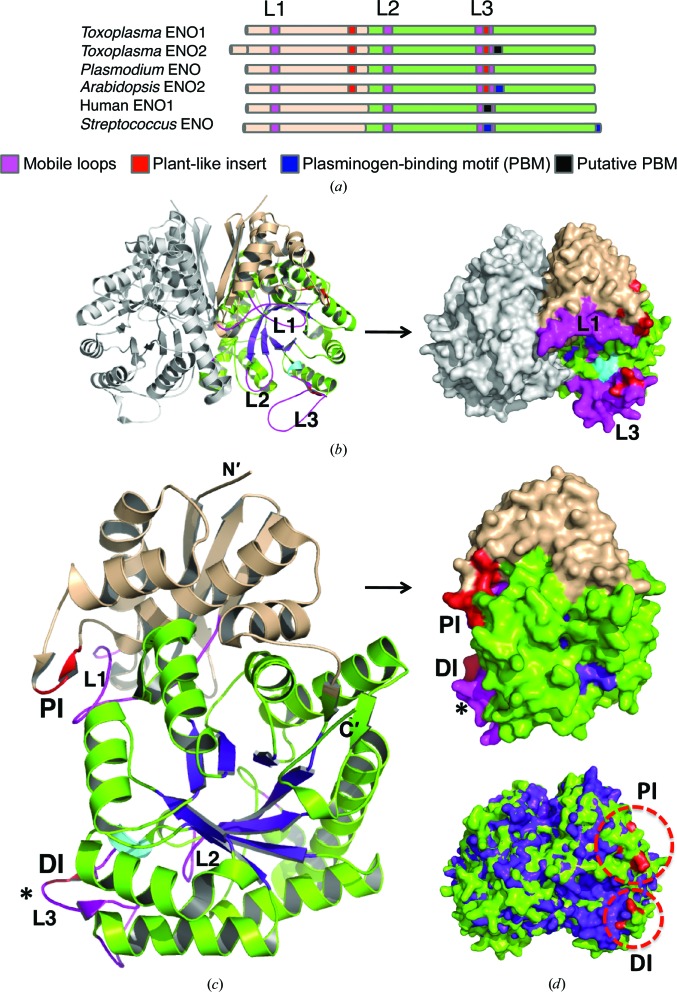
Structure of bradyzoite TgENO1. (*a*) The domain topology of TgENO1 is compared with selected enolases. (*b*) TgENO1 dimer. (*c*) TgENO1 monomer. The N-terminal domain (in wheat), C-terminal domain (in green), canonical TIM barrel (TIM), three mobile loops (L1–L3; magenta) and plant-like insertions (PI and DI; red) are shown. The *Plasmodium*-derived plasminogen-binding motif (asterisk) is located on surface loop 3 in TgENO2 but not in TgENO1. (*d*) Plant-like insertions (circled) are labelled red in TgENO1 (green) and superposed with human ENO1 (purple; PDB entry 2psn; J. K. Hyo, J. K. Seung, J. C. Sang & J. Suk-Kyeong, unpublished work) which does not contain the plant-like insertion.

An apo TgENO1 crystal structure was determined in space group *I*4 to 2.75 Å resolution (Table 1 ▶). The crystallographic asymmetric unit contained three nearly identical TgENO1 homodimers. Similar to the related enolases, each of the 436-amino-acid TgENO1 subunits folds into a small N-terminal domain (residues 1–150) and a larger catalytic C-terminal domain (residues 151–444) (Figs. 1 ▶ and 2 ▶ *c*). The N-terminal domain constitutes a three-stranded β-sheet with four α-helices, while the C-terminal domain forms a TIM-barrel catalytic domain (Fig. 2 ▶ *c*) that is conserved across members of the enolase superfamily (Gerlt *et al.*, 2012 ▶).

### 3.3. Plant-like insertions in *Toxoplasma* enolases   

Plant enolases contain pentapeptide [PI; EWGW(Y)C(S)] and dipeptide (DI; EK/DK, KQ) insertions. While absent in most yeast and animal enolases, these two insertions are present in both TgENO1 and TgENO2 (Figs. 1 ▶ and 2 ▶; Dzierszinski *et al.*, 2001 ▶; Harper & Keeling, 2004 ▶). A pair of deletion studies underscores the functional importance of these plant-like insertions. Deletion of the pentapeptide motif in *Plasmodium* enolase caused the enzyme to dissociate into monomers and resulted in a drastic ∼100-fold reduction in catalytic efficiency (Vora *et al.*, 2009 ▶). Deletion of one or both of the plant-like insertions in TgENO1 revealed that the insertions act synergistically to increase substrate affinity (Dzierszinski *et al.*, 1999 ▶). Two additional dipeptide insertions (ID at position 147 and EK at position 323) in TgENO1 have not been reported or analyzed for their potential function.

The TgENO1 structure reveals that the two plant-like enolase insertions are located on surface loops (Fig. 2 ▶). The larger pentapeptide insertion EWGYS forms a short β-strand (β5) between the α3 and α4 helices. The insertion produces a more positively charged surface potential at this N-terminal domain loop. The dipeptide insertion KQ, which is between β11 and β12, also increases the charged surface potential of loop L3 in the C-terminal domain, providing a possible site for binding to ligands that will negatively regulate the catalytic pocket. The uncharacterized dipeptide insertions at positions 147 and 323 are also located on surface loops.

**Figure 3:**
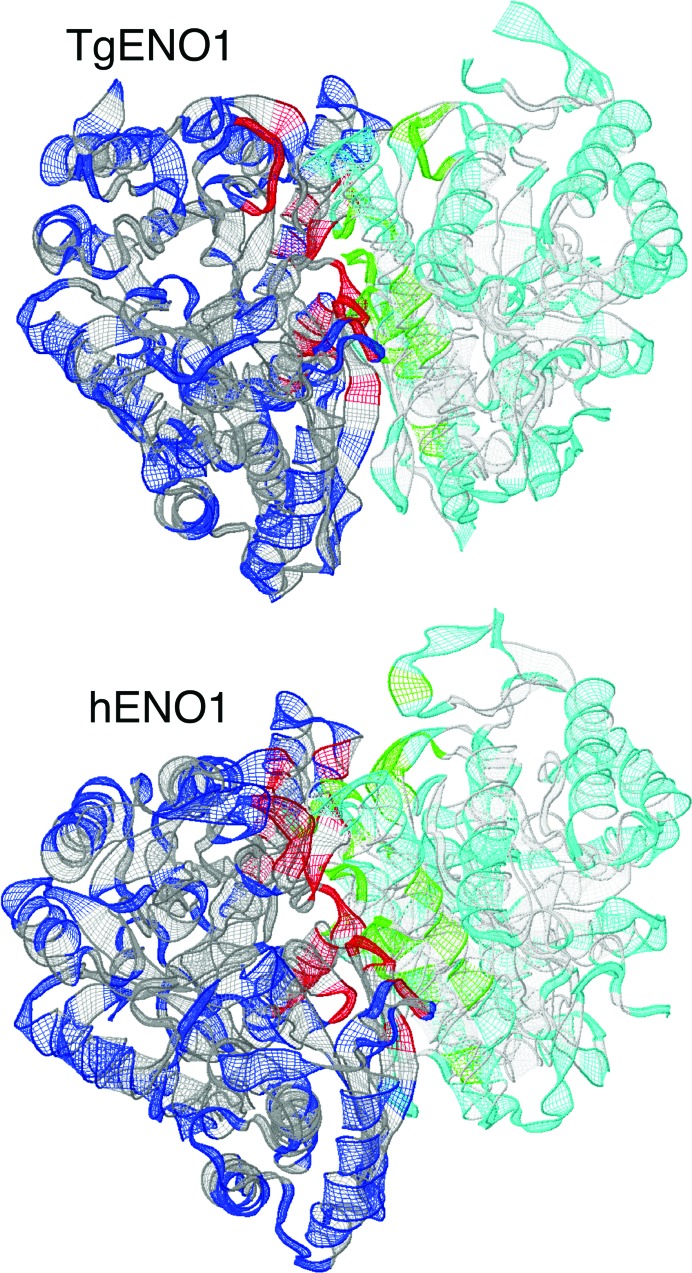
Enolase dimer interface. The subunit interfaces in *Toxoplasma* and human ENO1 dimers are illustrated in red and green. The *Toxoplasma* ENO1 dimer is formed by 33 hydrogen bonds, yielding an average surface area of 1891.7 Å^2^, as determined by *PISA* and 26 hydrogen bonds from *LigPlot*+ (Supplementary Fig. S1*a*), whereas the human ENO1 dimer interface contains 43 (*PISA*) and 31 (*LigPlot*+) hydrogen bonds (Supplementary Fig. S1*b*) with an average surface area of 2003.2 Å^2^.

The interaction of the N-terminal domain of one subunit with the C-terminal domain of the other subunit forms the TgENO1 dimer interface that buries 1891.7 Å^2^ and contains 33 hydrogen bonds and 15 salt bridges. This is comparable to the hENO1 dimer, which buries 2003.2 Å^2^ and contains 43 hydrogen bonds and 20 salt bridges as predicted using the *PISA* server (Xu *et al.*, 2008 ▶). Graphical illustrations (Fig. 3 ▶, Supplementary Fig. S1) of the dimer interface were analyzed using *LigPlot*+ v.1.3 (Laskowski & Swindells, 2011 ▶), which predicted the lower numbers of 26 and 31 hydrogen bonds for TgENO1 and hENO1, respectively, because of different geometric cutoffs (Supplementary Fig. S1). A comparison of bradyzoite TgENO1 and tachyzoite TgENO2 indicates a high level of conservation of hydrogen-bond-forming residues at the dimer interface. Since the pentapeptide is at some distance from the dimer interface, the monomeric state that results from deletion of the pentapeptide must be the consequence of substantial conformational changes (Vora *et al.*, 2009 ▶).

### 3.4. The bradyzoite TgENO1 active site   

**Figure 4:**
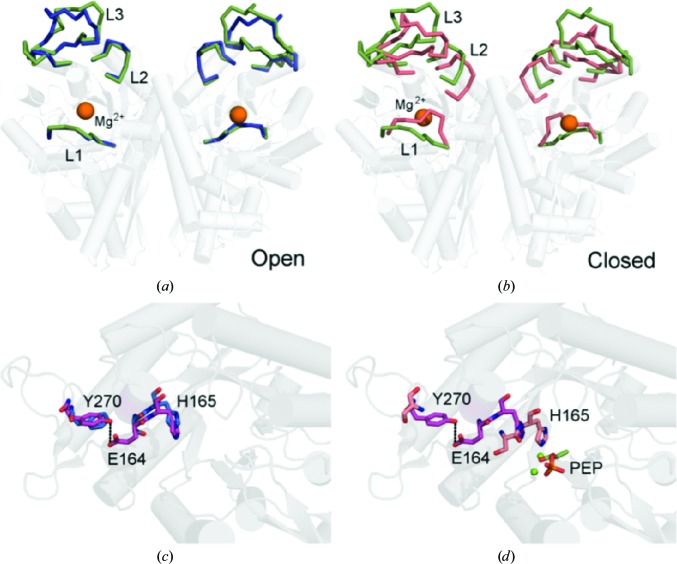
The TgENO1 active site. (*a*) Superposition of the TgENO1 dimer (green) with the ‘open-loop’ ScENO dimer (blue; PDB entry 1ebh; Wedekind *et al.*, 1995 ▶). The r.m.s.d. is 0.56 Å over 777 C^α^ atoms. (*b*) Superposition of the TgENO1 dimer with the ‘closed-loop’ hENO1 dimer (orange; PDB entry 3b97; Kang *et al.*, 2008 ▶). The r.m.s.d. is 0.68 Å over 645 C^α^ atoms. (*c*) A different perspective of the superposition in (*a*) highlights the hydrogen bond (dashed line) between the unique TgENO1 residue Glu164 in loop 2 and residue Tyr270 in loop 3. (*d*) Superposition of TgENO1 on the closed-loop PEP-bound complex (PDB entry 3ucd; Qin *et al.*, 2012 ▶) illustrates that L2 closure necessitates breakage of the Glu164–Tyr270 hydrogen bond and should allow His165 to hydrogen-bond to a PEP O atom.

Conversion from the tachyzoite stage to the bradyzoite stage is accompanied by a dramatic shift from oxidative to anaerobic glycolytic processes (Dzierszinski *et al.*, 2004 ▶; Tomavo, 2001 ▶). Potentially important in this transformation, bradyzoite TgENO1 exhibits a similar *K*_m_ value for 2-phospho-d-glycerate when compared with the tachyzoite TgENO2, but has a threefold lower *k*_cat_ (Dzierszinski *et al.*, 1999 ▶). The enolase active site is located in the central cavity formed by the C-termini of the β-strands and contains loop 1 (L1; residues 36–57), loop 2 (L2; residues 166–173) and loop 3 (L3; residues 258–282) (Figs. 1 ▶, 2 ▶ and 4 ▶). Previous studies have reported that these loops adopt an ‘open’ conformation in the unliganded and PEP-bound states, but undergo conformational changes to adopt a ‘closed’ conformation in 2-phospho-d-glycerate-bound and Mg^2+^-bound states (Lebioda *et al.*, 1989 ▶; Zhang *et al.*, 1997 ▶). L1 acts a ‘lid’ and closes the active site upon binding of substrate and cofactor using the residue Ser41/39 (TgENO1/human ENO1) in L1 by coordinating with the Mg^2+^ cofactor. The closed conformation facilitates protonation by His165/159 of the α carbon in 2-phospho-d-glycerate and the subsequent deprotonation by Lys355/345 to form a carbanion intermediate. Phosphoenolpyruvate is formed by elimination of hydroxide from carbon 3 by residue Glu217/211 and is then released as the three dynamic loops shift to an ‘open’ conformation. Superimposing TgENO1 with previously reported enolase structures shows the three loops in the apo TgENO1 structure assume an ‘open’ state (Figs. 4 ▶ *a* and 4 ▶ *b*).

While key catalytic and ligand-binding residues (TgENO1/human ENO1: His165/159, Glu174/168, Glu217/211, Lys355/345 and Lys406/396) are conserved, TgENO1 exhibits three atypical residues (Glu164, Leu176 and Gln380) in the active-site pocket in comparison to TgENO2 and other enolases (Fig. 1 ▶). Most notable in the bradyzoite TgENO1 is the presence of Glu164 at a position that is most commonly occupied by a serine in other enolases (Fig. 1 ▶). Ser164 is the N-terminal residue of L2 and interacts with the substrate/product phosphate group. In the bradyzoite TgENO1 structure, the Glu164 side chain points away from the active site and is within hydrogen-bonding distance of Tyr270 (Figs. 1 ▶, 4 ▶ *c* and 4 ▶ *d*). Tyr270 is also a unique residue that is commonly occupied by a phenylalanine in the critical L3. As the most prominent differentiating feature, Glu164 may account for the difference in *k*_cat_ between bradyzoite TgENO1 and tachyzoite TgENO2 by requiring a breakage of the Glu164–Tyr270 bond in repositioning the L2 and L3 to prime the catalytic pocket for the elimination reaction.

### 3.5. Surface potentials of transcription regulatory enolases   

**Figure 5:**
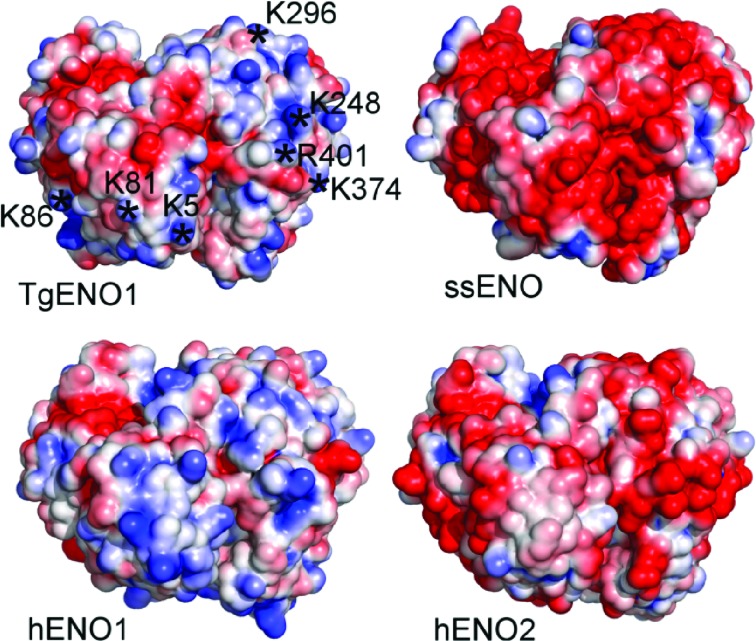
Surface electrostatic potentials of enolases. The cytosolic ssENO and hENO2 and the transcription factors TgENO1 and hENO1 are shown in surface representation and coloured by charge. Blue represents positive charge and red negative charge. Asterisks denote identical and/or conserved positively charged residues in nuclear enolases (see Fig. 1 ▶). Electrostatics were calculated and visualized using the *APBS* plugin in *PyMOL*. ssENO, *S. suis*, PDB entry 4ewj (Lu *et al.*, 2012 ▶). hENO2, *Homo sapiens*, PDB entry 1te6 (Chai *et al.*, 2004 ▶).

Canonical DNA-binding motifs (helix–turn–helix, basic helix–loop–helix, helix–loop–helix, leucine zipper, zinc finger and HMG box) are not identifiable in TgENO1 or TgENO2 or in human and plant transcription regulatory enolases. However, a comparison of calculated surface electrostatic potentials (Fig. 5 ▶) indicates that the nuclear enolases (hENO1 and TgENO1) tend to have a higher degree of positive potential than the cytoplasmic enolases (hENO2, *Streptococcus pneumoniae* ENO1). The positively charged residues (lysine and arginines) on the surface of bradyzoite TgENO1 are conserved in tachyzoite TgENO2 (Fig. 1 ▶), which is also a nuclear factor that activates gene promoters (Mouveaux *et al.*, 2014 ▶). The positive surface charge of *Toxoplasma* enolases is consistent with protein interfaces that bind to the negatively charged surface of DNA (Nadassy *et al.*, 1999 ▶). Thus far, our extensive efforts to co-crystallize complexes of TgENO1 and TTTTCT motif-containing DNA to structurally validate the positive surface patches as a novel nucleic acid-binding surface have been unsuccessful.

### 3.6. Evidence for specific TgENO1–DNA interactions   

**Figure 6:**
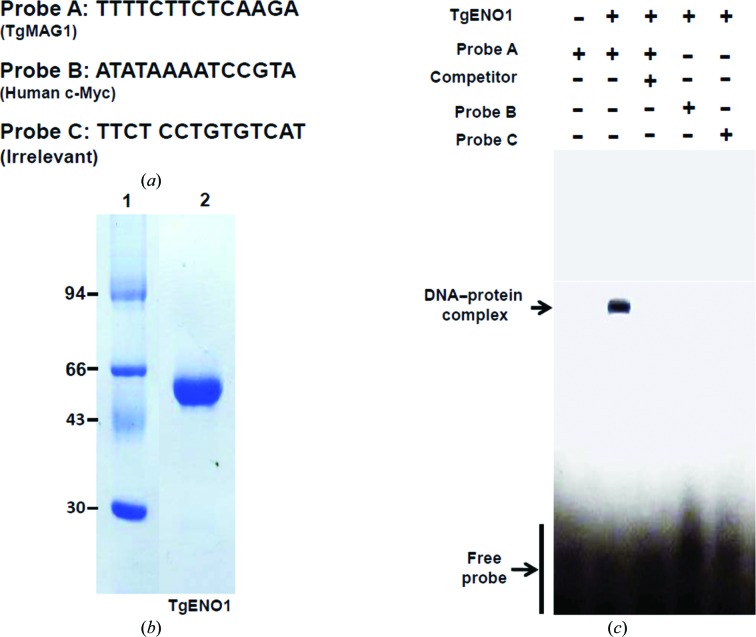
*In vitro* interaction of TgENO1 with the TgMAG1 promoter sequence. (*a*) Oligonucleotide probes were designed for the TTTTCT motif in the bradyzoite TgMAG1 promoter, the TATA box in the human c-­Myc promoter and a negative random sequence. (*b*) SDS–PAGE of purified recombinant TgENO1. Lane 1 contains molecular-weight marker (labelled in kDa). (*c*) Electrophoretic mobility shift assay of biotinylated TgENO1 incubated with probes and detected for retardation mobility of the DNA–protein complex. The TgENO1–TgMAG1 complex (second lane) was competed with an unlabelled probe prior to incubation with labelled TGMAG1 probe (third lane).

In the absence of crystallographic data, we have however further confirmed the role in transcription of the bradyzoite TgENO1 by testing the physical interaction of this bradyzoite isozyme with the TTTTCT motif that is present in promoters of a repertoire of genes that are tightly regulated during stage conversion, including the cyst matrix antigen (MAG1) gene (Mouveaux *et al.*, 2014 ▶). We tested binding of the recombinant TgENO1 to this TTTTCT and other control DNA motifs (Fig. 6 ▶ *a*). The gel retardation shown in Fig. 6 ▶(*c*) demonstrates that the recombinant TgENO1 protein specifically interacts with a TgMAG1 probe containing the TTTTCT motif. In contrast, TgENO1 protein did not bind to a c-Myc motif that contains the canonical TATA box in probe B that is known for its binding to human or plant enolases (Lee *et al.*, 2002 ▶) or to the unrelated probe C (Fig. 6 ▶ *c*). Additionally, PCR of the control regions are negative (data not shown). Thus, TgENO1 specifically interacts with a specific DNA motif present in a *T. gondii* gene promoter.

### 3.7. TgENO1 specifically targets the MAG1 promoter *in vivo*   

**Figure 7:**
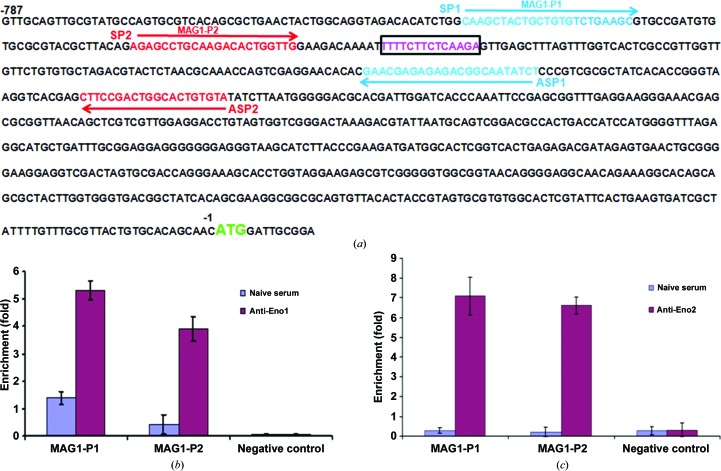
*In vitro* interaction of TgENO1 and TgENO2 with the bradyzoite TgMAG1 promoter as shown by chromatin immunoprecipitation (ChIP) analyses. (*a*) Upstream and downstream primers of the TTTTCT motif in TgMAG1 for PCR of the ChIP products. (*b*) Polyclonal antibodies to bradyzoite-specific TgENO1 precipitated ChIP fragments from the TgMAG1 promoter. (*c*) Polyclonal antibodies to tachyzoite-specific TgENO2 also pulled down ChIP fragments from TgMAG1 promoter.

Having shown that TgENO1 binds to a specific DNA motif by gel retardation *in vitro*, we focused on validating its binding to the MAG1 gene promoter *in vivo*. We showed that the rabbit polyclonal antibodies specific to TgENO1 pulled down the MAG1 promoter from chromatin extracts of intracellular parasites (Fig. 7 ▶ *b*) using two distinct pair of primers that span the TTTTCT motif (Fig. 7 ▶ *a*). In addition, the MAG1 promoter was also similarly pulled down with the polyclonal anti-ENO2 antibodies and the same pair of primers (Fig. 7 ▶ *c*). These results indicate that both bradyzoite-specific TgENO1 and tachyzoite-specific TgENO2 are capable of binding to the TgMAG1 gene promoter, suggesting a possible role of nuclear enolases in the control of gene expression in *T. gondii*. This proposed role is further confirmed by targeted disruption of TgENO1, which resulted in changes in the transcripts of nuclear genes and a reduction in the brain-cyst burden in chronically infected mice (Mouveaux *et al.*, 2014 ▶). This moonlighting enzyme is thus proposed to play a transcriptional regulatory role in brain-cyst development.

### 3.8. Evolutionary history of transcription regulatory enolases   

Identification of TgENO1 and TgENO2 brings the number of enolases with verified transcription-regulatory properties to four, those from *Toxoplasma* (TgENO1 and TgENO2), *Arabidopsis* (AtENO2/LOS2) and human (hENO1/hMPB1). To evaluate the evolutionary relationship between the known transcription regulatory enolases, maximum-likelihood phylogenetic analysis was performed (Supplementary Fig. S2) using 157 enolase amino-acid sequences that represent 24 phylogenetic phyla. The resulting bootstrap consensus tree reveals a complex evolutionary history and is in general agreement with earlier studies of the distribution of enolase insertions in that no single event can explain enolase phylogeny (Harper & Keeling, 2004 ▶). Enolase evolutionary history is likely to be owing to a combination of lateral gene transfer, paralogy and recombination. Most relevant to this study is that the DNA-binding enolases do not share a common origin. Although the sample size is limited to only four, preliminary analysis suggests that the properties of nuclear localization, DNA-binding and transcriptional regulation are likely to be the product of convergent evolution. The low conservation of positively charged residues that are proposed to indicate the DNA-binding surface (Fig. 7 ▶) between TgENO1, plant ENO2 and human ENO1/MPB1 (Fig. 1 ▶) supports the suggested convergent evolution. Future studies will indicate whether such functional convergence can reveal critical structure–function relationships between the parasite and mammalian host, delineating a specific localized region and function for targeting the parasite enolase without unintended effects on the human enzymes.

In conclusion, this study provides the second structural analysis of bradyzoite proteins that specifically examines the multiple and differential functions of enolases, which in turn may reveal variable windows for targeting drugs against both acute and chronic infections by the brain parasite *Toxoplasma*.

## Supplementary Material

PDB reference: *Toxoplasma gondii* enolase 1, 3otr

Supporting Figures.. DOI: 10.1107/S1399004714026479/mn5082sup1.pdf

## Figures and Tables

**Table 1:** Data-collection and refinement statistics Values in parentheses are for the highest resolution shell.

PDB code	3otr
Data collection	
Wavelength ()	1.07809
Temperature (K)	100
Space group	*I*4
Unit-cell parameters (, )	*a* = *b* = 323.6, c = 66.8, = = = 90
Resolution range ()	30.02.75 (2.802.75)
No. of reflections	90781 (4489)
*R*_merge_	0.113 (0.543)
Completeness (%)	100.0 (100.0)
*I*/(*I*)	11.6 (2.6)
Multiplicity	4.0 (4.0)
Wilson *B* factor (^2^)	58.4
Refinement	
Resolution range ()	29.552.75 (2.822.75)
No. of reflections	86109 (6280)
*R*_work_/*R*_free_	0.171/0.219 (0.257/0.327)
Protein molecules/atoms	6/20363
Solvent atoms	584
Mean temperature factor (^2^)	37.59
Coordinate deviation	
R.m.s.d., bonds ()	0.007
R.m.s.d., angles ()	1.32
Ramachandran plot	
Most favoured (%)	88.8
Allowed (%)	10.9
Generously allowed (%)	0.3
Disallowed (%)	0.0
