# Supplementary material for: The structure of bradyzoite-specific enolase from Toxoplasma gondii reveals insights into its dual cytoplasmic and nuclear functions
Source: Acta Crystallogr D Biol Crystallogr. 2015 Feb 26;71(Pt 3):417–26. doi: 10.1107/S1399004714026479 (PMC4356359; doi:10.1107/S1399004714026479)

BIOLOGICAL CRYSTALLOGRAPHY

Volume 71 (2015)

Supporting information for article:

The structure of bradyzoite-specific enolase from *Toxoplasma gondii* reveals insights into its dual cytoplasmic and nuclear functions

Jiapeng Ruan, Thomas Mouveaux, Samuel H. Light, George Minasov, Wayne F. Anderson, Stanislas Tomavo and Huân M. Ngô

Supplement 1A. Hydrogen bonds of *Toxoplasma* ENO1 dimer interface.

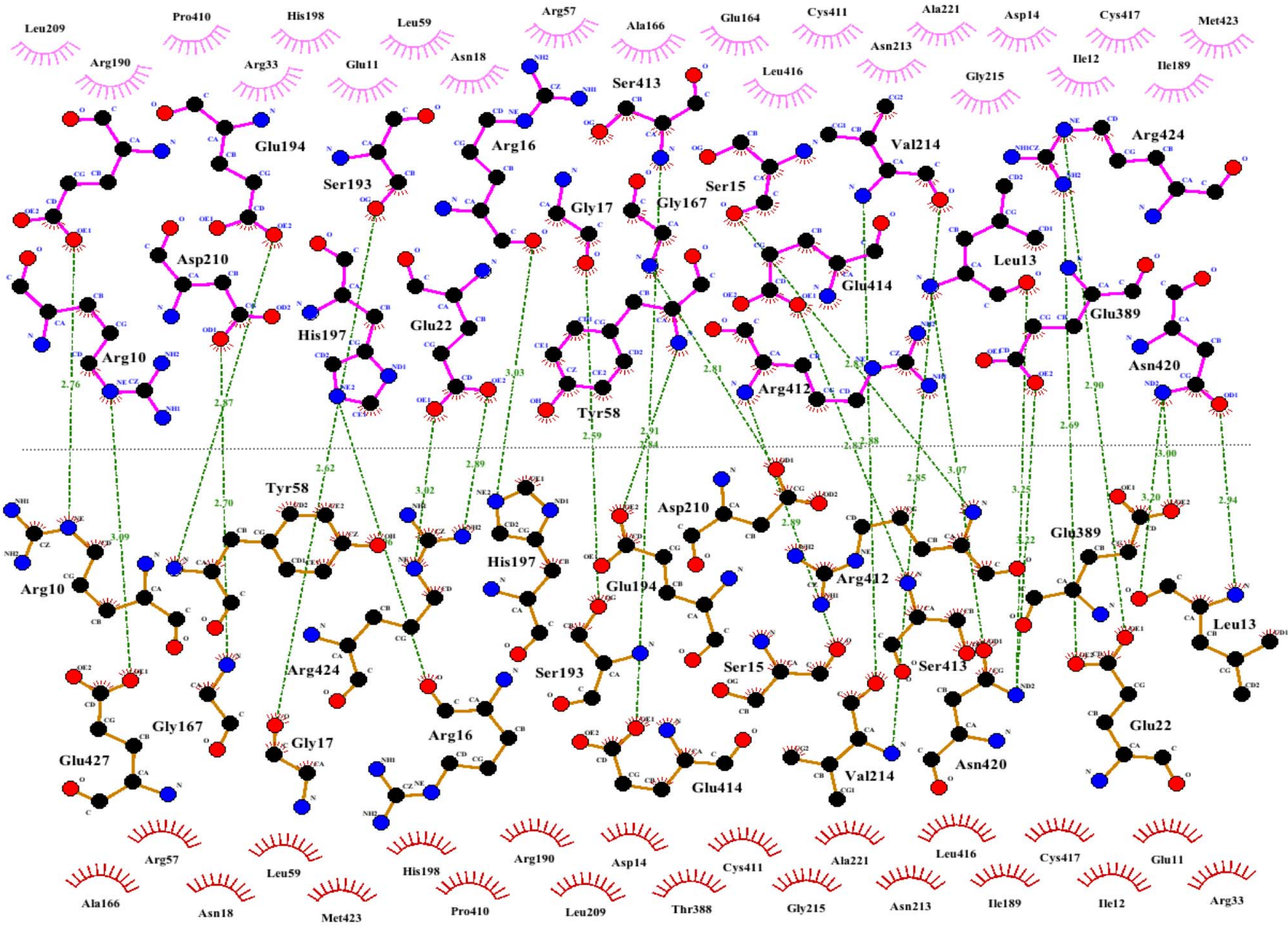

Supplement 1B. Hydrogen bonds of human ENO1 dimer interface.

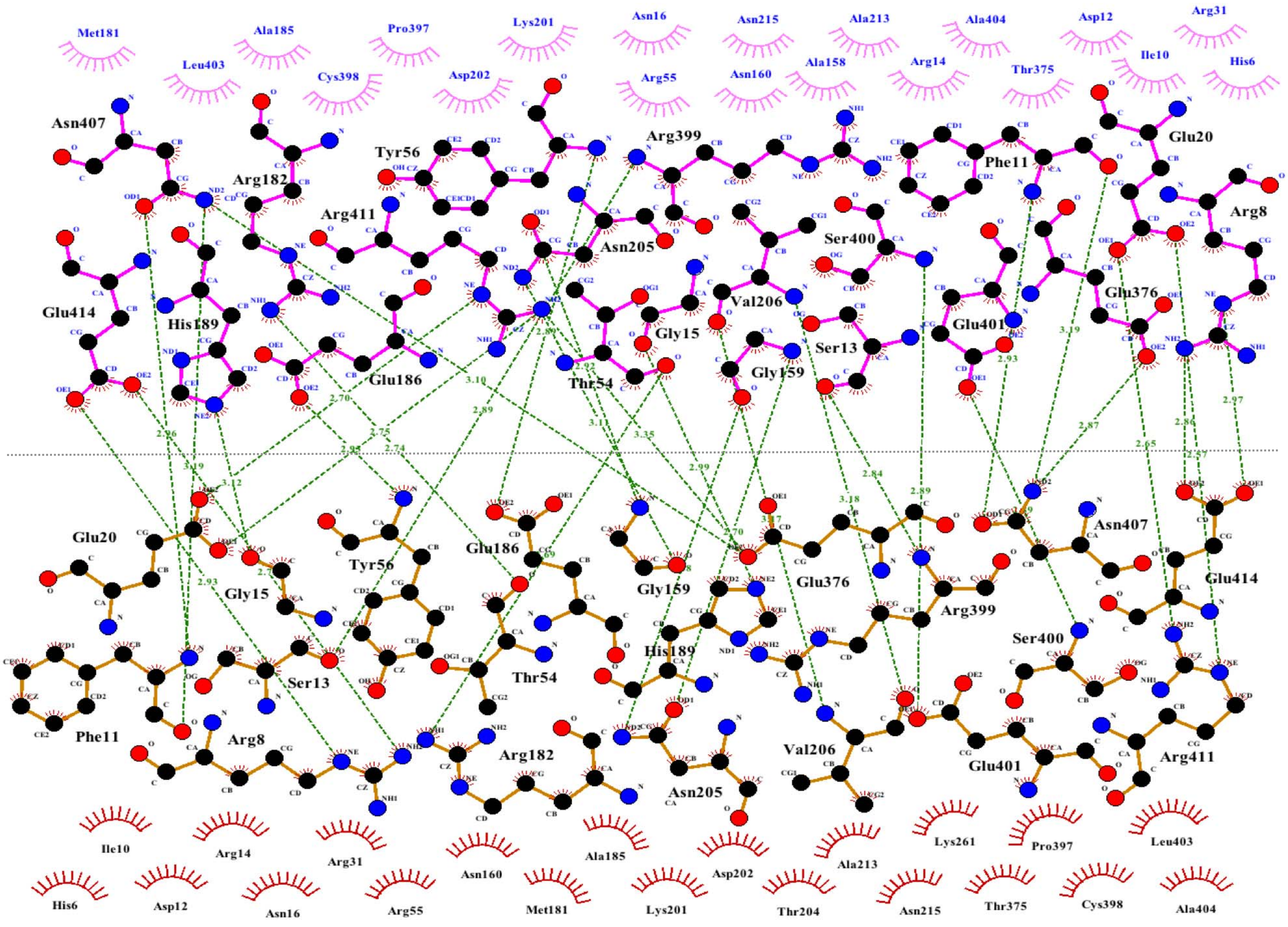

Supplement 2. Convergent evolution of DNA binding enolases. Phylogram of 157 taxa from 24 phyla is constructed by Maximum Likelihood method that is based on the JTT matrix-based model. The bootstrap consensus tree is inferred from 1000 replicates. Evolutionary analyses were conducted in MEGA5.

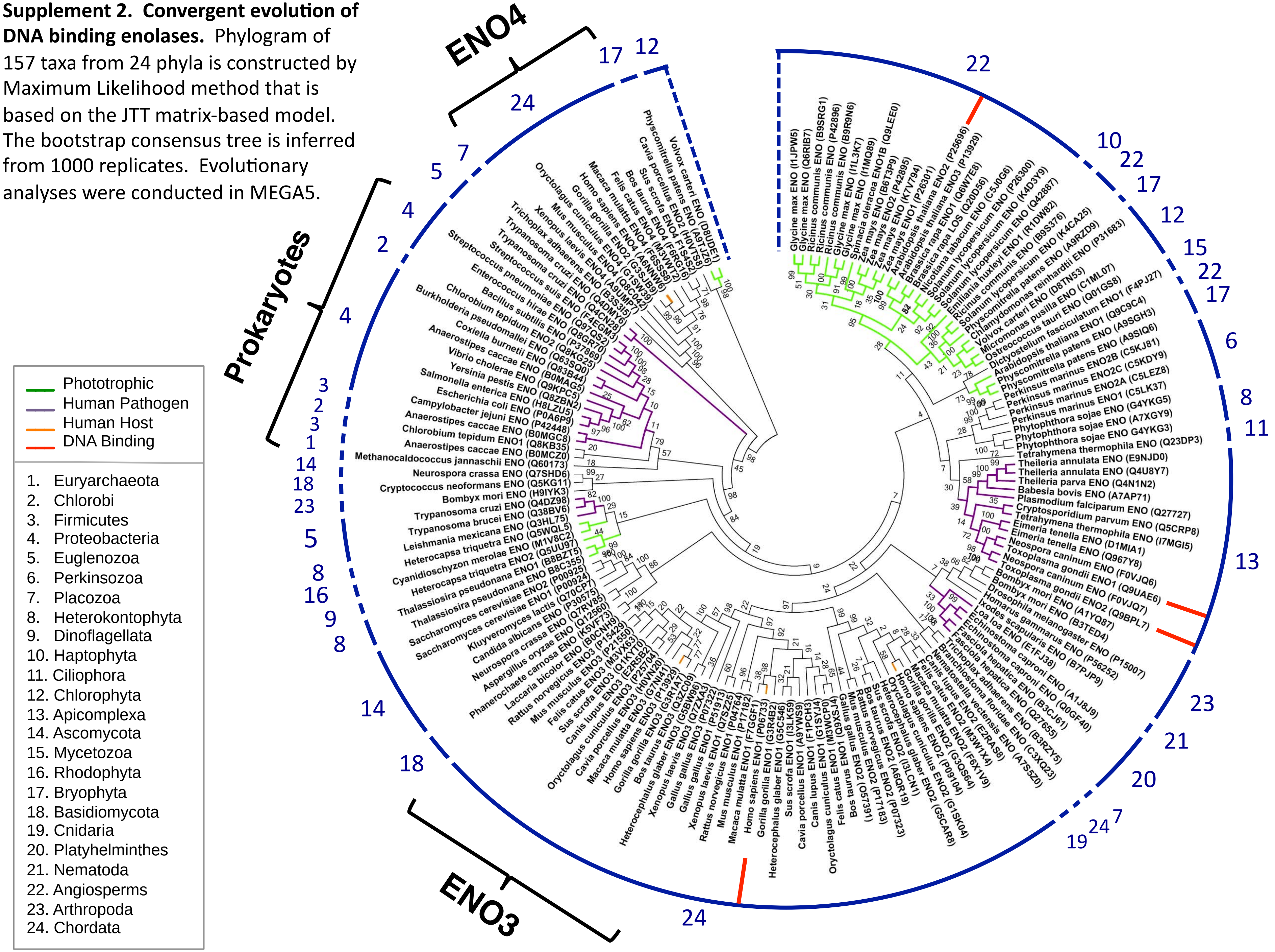

Supplement: Supplementary file 1 [file d-71-00417-sup1.pdf]
